# A Grandmother's Advice to Young Mothers on the Physical Education of Children

**Published:** 1835-07-01

**Authors:** 


*A Grandmother's Advice to Young Mothers on the Physical Education of Children.* By M. J., Countess Dowager Mountcashell. *Revised and augmented by the Author.* London, 1835. Small 8vo. pp. 383.

This is a hygienic treatise of very extraordinary merit, and is evidently the offspring of a clear head, long experience, and sound medical reading. The late Andrea Vaccà, of Pisa, encouraged the noble author in her undertaking, and however fastidious this eminent surgeon may have been, he had no reason to be ashamed of his pupil.

Those who have seen and sighed over the stupid manner in which new-born infants are commonly washed, will be delighted with the following passage.

"The washing of a new-born infant ought not to give it pain; and in fact, if performed in a convenient manner, would rather be likely to occasion agreeable than disagreeable sensations. The best method I have ever seen is that employed at Vienna, where they have for this purpose a wooden vessel made in the form of a long oval tub, of dimensions proportioned to the use for which it is designed. It is filled with tepid water, (in which may be mixed a little brandy or soap if thought necessary,) and in this bath the infant is placed by the midwife, who supports it under the back of the head with one hand. After it has remained three or four minutes in the water, which should be in sufficient quantity to make it float, she rubs it tenderly all over with a soft sponge, and then dries it gently with a warm napkin. An attendant should be ready to cover the child the moment it is lifted out of the water, and care should be taken to put the napkin *first over* (rather than *under*) its body, as that will prevent it from feeling a painful sensation by the impression of the comparatively cold air. It is scarcely necessary to add that this first washing should take place in a warm room, with all doors and windows closed.

"Besides the wooden vessel above mentioned, there is also prepared a large square cushion, which is used in dressing the child; and this is laid on a table, which is extremely convenient for the person employed in this office. It is filled with chopped straw in such a manner as to be pliable to the weight of the infant, and may be pressed into any form that is commodious. On this the child is laid when taken out of the bath, a warm napkin having been previously spread over it; and, after being well dried in a position which gives no fatigue, the child is thus dressed without having its arms pulled about unnecessarily, or being forced into the unnatural posture of sitting. The clothes of the child are made to fasten behind, and so shaped as to cover the breast and arms; a necessary precaution in cold climates, and an advantage in all. Indeed I have been convinced by repeated observation in various countries, that children who have their bosoms and arms covered for the first two years, are not subject to those severe coughs and inflammations of the lungs which are, during the time of teething, fatal to so many in the British Islands.

"Another thing in the dress of infants at Vienna (as well as in many other places on the Continent) deserves also to be imitated; which is, that not one pin is employed in their clothing, every article that requires to be fastened having strings; and the person who ties them turns the child on its side as it lies on the straw cushion, so that it suffers no inconvenience. Some English nurses may, perhaps, say that it is impossible to dress a child entirely without pins: but what is done in one place may be done in another, and I recommend nothing of which I have not witnessed the advantage.

"As long as it is necessary to have the child swathed, this is done with peculiar ease by laying it on its back on the straw cushion, holding up the feet with one hand (or making an attendant do so) and rolling the bandage round it with the other. The only pressure that should be made on the body of an infant, is that which is required for some time after the division of the umbilical cord; and which is often a beneficial part of the clothing, after it has ceased to be the necessary bandage of a wound. The swathe should be made of soft linen doubled, without seam or hem, and should have two strings at one end, long enough to go once round the body and to tie. It should be rather more than an inch and a half in breadth and two or three yards in length, according to the size of the child. When this is applied by a judicious hand, it is in many cases advantageous, especially where there has been any griping or looseness, both on account of warmth and of gentle pressure on the bowels; and might in some instances be continued with good effect for three months, or even longer if the weather be cold: for no diminution in a child's clothing should ever take place except in a warm season." (P. 35.)

The learned doctors who have written of late against stays, will be glad that they have an ally in our author. An enthusiast might almost suppose that there was some chance of this absurdity disappearing before the year 2000, when it is attacked by one who belongs to the aristocracy of rank as well as of intellect, for those who will not listen to Soemmerring may be convinced by a Countess.

"But the worst of all pressures is what is frequently inflicted on the bodies of female children, by that most detrimental of fashions, the use of stays,—the origin of a thousand deformities and diseases, and the cause of many fatal accidents. Were it even true that an excessively small waist was a necessary part of beauty, and that great sacrifices ought to be made for the acquisition of it, we should first consider how far this mode of squeezing the stomach and bowels is likely to have the desired effect; or whether it is worth while, for the doubtful chance of obtaining this end, to run the risk of producing certain ugliness, by crookedness and bad health. I have very good reason for believing that this mode of acquiring a slender shape does not always succeed; and, on the contrary, I have known many instances of clumsy girls, whose forms were entirely left to nature, growing up with much smaller waists than others who had been subjected to the tortures of fashion.

"I well recollect in my youth to have heard certain individuals blamed, extremely, for their very injudicious and careless conduct towards their daughters, who were doomed, by many prophetic voices, 'to grow up as thick round the body as kitchen-maids;' and I have afterwards seen those very young women, who had been so pitied for the cruel neglect of their parents, with more slender waists, and (what were then called) finer shapes than any of their neighbours, who had enjoyed all the advantages of being squeezed and tormented from their infancy.

"Beauty is by no means to be neglected; but it cannot exist without proportion; and if a girl be so formed as to have broad shoulders and broad hips, (as many handsome women are,) surely nothing is so calculated to destroy the symmetry of her shape as to pinch in her waist until it is as small as her arms. Besides, let it be remembered, that whatever hurts the health must produce ugliness in a greater or less degree; and all persons who know anything of medicine, can have but one opinion on the subject of *tight lacing*." (P. 317.)

We cannot bestow the same praise on the therapeutic part of the work, in which Lady Mountcashell endeavours to teach mothers to treat the diseases of their children. It is well written, but the thing cannot be done. This clever attempt at the impossible is defended in the following passage.

"To many persons it may appear, that in saying so much about the cure of diseases, the author transgresses the bounds she had prescribed to herself; but on examination it will be found that this is not the case. She would always recommend those whose circumstances enable them to procure the assistance of a *really skilful* physician, to profit by his experience even in trifling maladies; but as this book is designed for mothers in every rank of society, many of whom live at a distance from medical aid, and others only on extraordinary occasions can afford the expense attendant on having *good* advice, it is but just to point out to such persons the means of curing slight indispositions, and of retarding (perhaps removing) danger in severe maladies; to mark the period when the aid of a physician must, if possible, be obtained, and to prevent the incalculable evils which continually occur in consequence of following the advice of ignorant pretenders." (P. 85, *Note.*)

To this we would reply, that those who cannot get first-rate advice, must be content with second, or third, or fifth-rate; but an anxious mother, with all her notions of physic gathered from a small manual, and her own child for her first patient, could scarcely be estimated by the depreciatory phraseology in ordinary use. Her medical tact, if she ever had any, would be in such a state of homœopathic dilution, of extreme proximity to nothing, that, to express it, we must adopt the notation of Hahnemann, and call her an X, or $(1,000,000)^{10}$th rate practitioner.

The other part of the work, however, containing the prophylactic regimen, is so singularly excellent, so remarkable for its brilliant good sense, female tact, and a certain elasticity of style, that we would suggest to the benevolent author that it should be separately printed at a very low price, that it might obtain the circulation which it deserves.

